# Distribution of gut microbiota across intestinal segments and their impact on human physiological and pathological processes

**DOI:** 10.1186/s13578-025-01385-y

**Published:** 2025-04-16

**Authors:** Ke Yang, Guangqin Li, Qihong Li, Wei Wang, Xu Zhao, Nan Shao, Hui Qiu, Jing Liu, Lin Xu, Juanjuan Zhao

**Affiliations:** 1 The First Clinical Institute, Zunyi Medical University, Zunyi, 563000 China; 2 Key Laboratory for Cancer Prevention and treatment of Guizhou Province, Zunyi Medical University, Zunyi, 563000 Guizhou China; 3 Guizhou University Medical College, Guiyang, 550025 Guizhou China; 4 Department of Immunology, Zunyi Medical University, Zunyi, 563000 Guizhou China

**Keywords:** Gut microbiota, Intestinal segments, Distribution characteristics, Physiological functions, Pathological processes

## Abstract

In recent years, advancements in metagenomics, metabolomics, and single-cell sequencing have enhanced our understanding of the intricate relationships between gut microbiota and their hosts. Gut microbiota colonize humans from birth, with their initial composition significantly influenced by the mode of delivery and feeding method. During the transition from infancy to early childhood, exposure to a diverse diet and the maturation of the immune system lead to the gradual stabilization of gut microbiota's composition and distribution. Numerous studies have demonstrated that gut microbiota can influence a wide range of physiological functions and pathological processes by interacting with various tissues and organs through the gut-organ axis. Different intestinal segments exhibit unique physical and chemical conditions, which leads to the formation of vertical gradients along the intestinal tract: aerobes and facultative aerobes mainly live in the small intestine and anaerobic bacteria mainly live in the large intestine, and horizontal gradients: mucosa-associated microbiota and lumen-associated microbiota. In this review, we systematically summarize the distribution characteristics of gut microbiota across six intestinal segments: duodenum, jejunum, ileum, cecum, colon, and rectum. We also draw a conclusion that gut microbiota distributed in different intestinal segments affect the progression of different diseases. We hope to elucidate the role of microbiota at specific anatomic sites within the gut in precisely regulating the processes of particular diseases, thereby providing a solid foundation for developing novel diagnostic and therapeutic strategies for related diseases.

## Introduction

Gut microbiota (GM) refers to the intricate and diverse community of microorganisms inhabiting the gastrointestinal tract of humans and animals. This microbial ecosystem is one of the most complex in the human body, comprising bacteria, archaea, viruses, and eukaryotic microbes. The sheer number of GM and their genes in humans far surpasses that of human cells and their genomes, rendering GM the “second largest gene pool” in humans [1, 2]. This vast genetic reservoir contributes to the microbiota's remarkable complexity, diversity, and resilience, which are crucial for maintaining host health and facilitating various biological processes.

The GM plays a pivotal role in numerous aspects of human physiology. It is essential for the digestion and fermentation of dietary fibers, the synthesis of essential vitamins (such as vitamin K and certain B vitamins), and the metabolism of bile acids and xenobiotics. Additionally, GM is integral to the development and modulation of the host immune system, helping to maintain immune homeostasis and protect against pathogenic microorganisms. Beyond these fundamental roles, emerging research has revealed the influence of GM on more intricate mechanisms, including neuroendocrine regulation and behavior, highlighting the microbiota-gut-brain axis [3]. Dysbiosis, or the imbalance of gut microbial communities, has been implicated in a wide array of clinical conditions. These include metabolic disorders such as obesity and type 2 diabetes, inflammatory diseases like Crohn's disease and ulcerative colitis, cardiovascular diseases, and even neurodegenerative diseases like Parkinson's and Alzheimer's. The precise mechanisms by which GM influences these conditions are an area of active investigation, underscoring the importance of understanding the spatial distribution and functional roles of microbiota within the gut.

The human gastrointestinal tract is a dynamic environment characterized by distinct anatomical regions, each providing unique niches that shape microbial colonization and activity. The gut can be broadly divided into the upper (duodenum and jejunum) and lower (ileum, cecum, colon, and rectum) sections, each with specific physiological functions and varying conditions such as pH levels, oxygen availability, and nutrient gradients [4]. These variations create both vertical gradients along the length of the intestine and horizontal gradients between the lumen and the mucosal surfaces within the same intestinal segment [5, 6]. For instance, the oxygen concentration decreases from the proximal to the distal gut, influencing the predominance of aerobic versus anaerobic bacteria.

Site-specific interactions between GM and the host are critical in determining the functional outcomes of the microbial communities. For example, in the ileum, bacteria such as *Bacteroides* and* Prevotella* participate in the enterohepatic circulation of bile salts through dehydroxylation processes, which in turn affect bile salt reabsorption and lipid metabolism [7]. In the colon, a more anaerobic environment supports the growth of* Firmicutes* and *Bacteroidetes*, which are key players in fermenting complex carbohydrates and producing short-chain fatty acids (SCFAs) like butyrate, propionate, and acetate. These SCFAs serve as energy sources for colonocytes, regulate immune responses, and influence systemic metabolism [8]. Understanding the spatial distribution and functional specialization of GM across different gut regions is essential for elucidating the mechanisms underlying their roles in health and disease. Variations in microbial composition and activity can lead to differential impacts on host physiology, making it crucial to consider anatomical specificity in both research and therapeutic interventions.

**Fig. 1:**
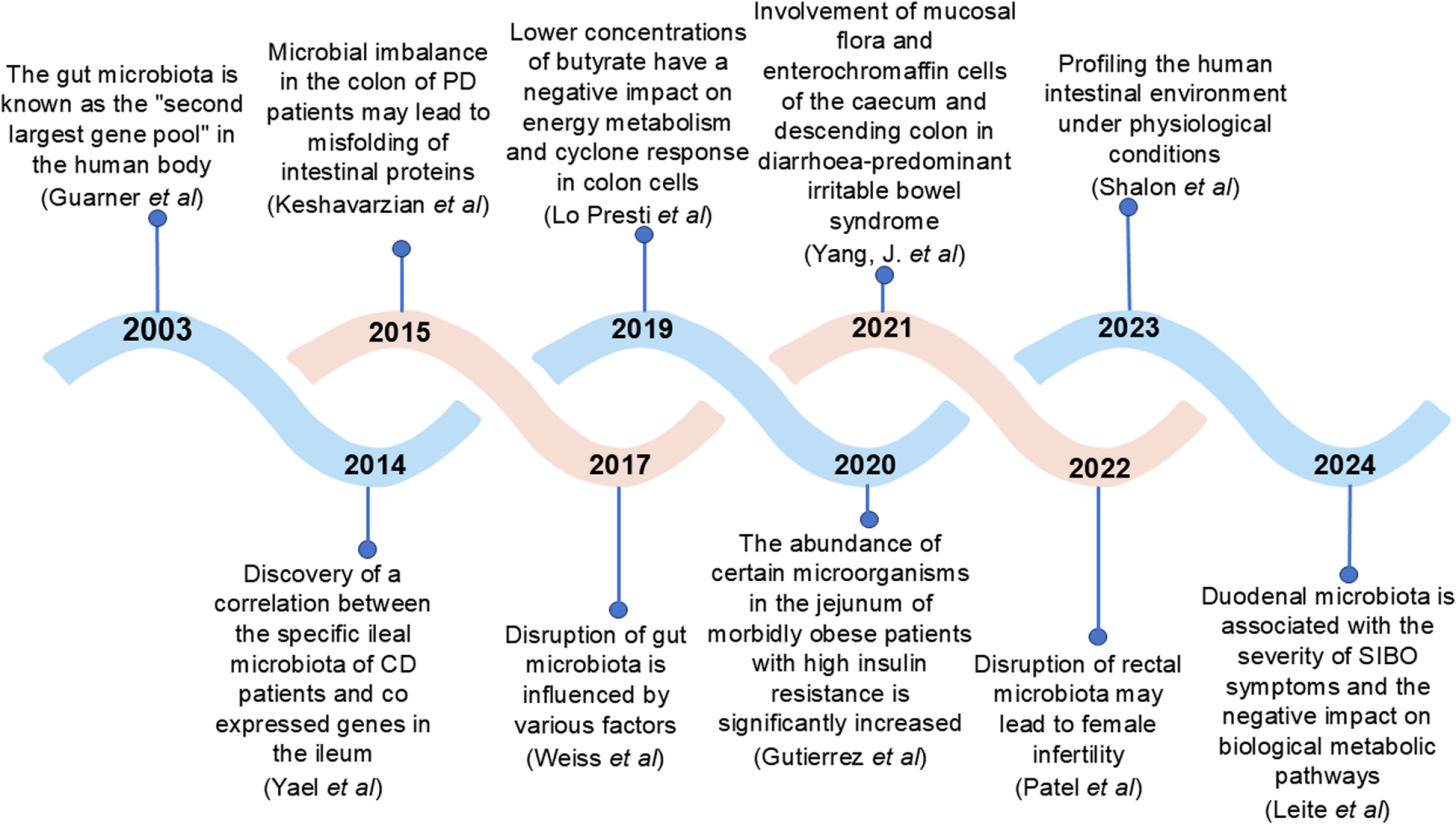
Research findings on gut microbiota

This review aims to elucidate the distribution characteristics of GM across different anatomic sites of the gut, including the duodenum, jejunum, ileum, cecum, colon, and rectum.We will explore the distinct roles that microbiota play in each of these regions, particularly in relation to physiological processes and pathological conditions. By summarizing the current understanding of segment-specific microbiota and their interactions with the host, we seek to highlight the unique contributions of each gut region to overall health and disease states (Fig. 1). Furthermore, this review will discuss the implications of these findings for the development of innovative diagnostic and therapeutic strategies targeting GM, thereby advancing the potential for precision medicine approaches in managing diverse diseases.

## Distribution characteristics of human gut microbiota

Dozens of bacterial phyla have been identified in the human GM. However, in most healthy individuals, GM primarily consists of six dominant bacterial groups: *Bacteroidota, Firmicutes, Actinomycetota, Proteobacteria, Verrucomicrobia, and Fusobacteria* [9, 10]. Numerous studies have demonstrated that while GM composition remains relatively stable at the phylum level, significant temporal and spatial variations exist at the species or genus level. The disruption of gut microbiota is influenced by various factors, which can be roughly divided into life cycle stages with temporal differences and coexisting diseases with spatial differences, drug therapy, and dietary factors.

**Fig. 2:**
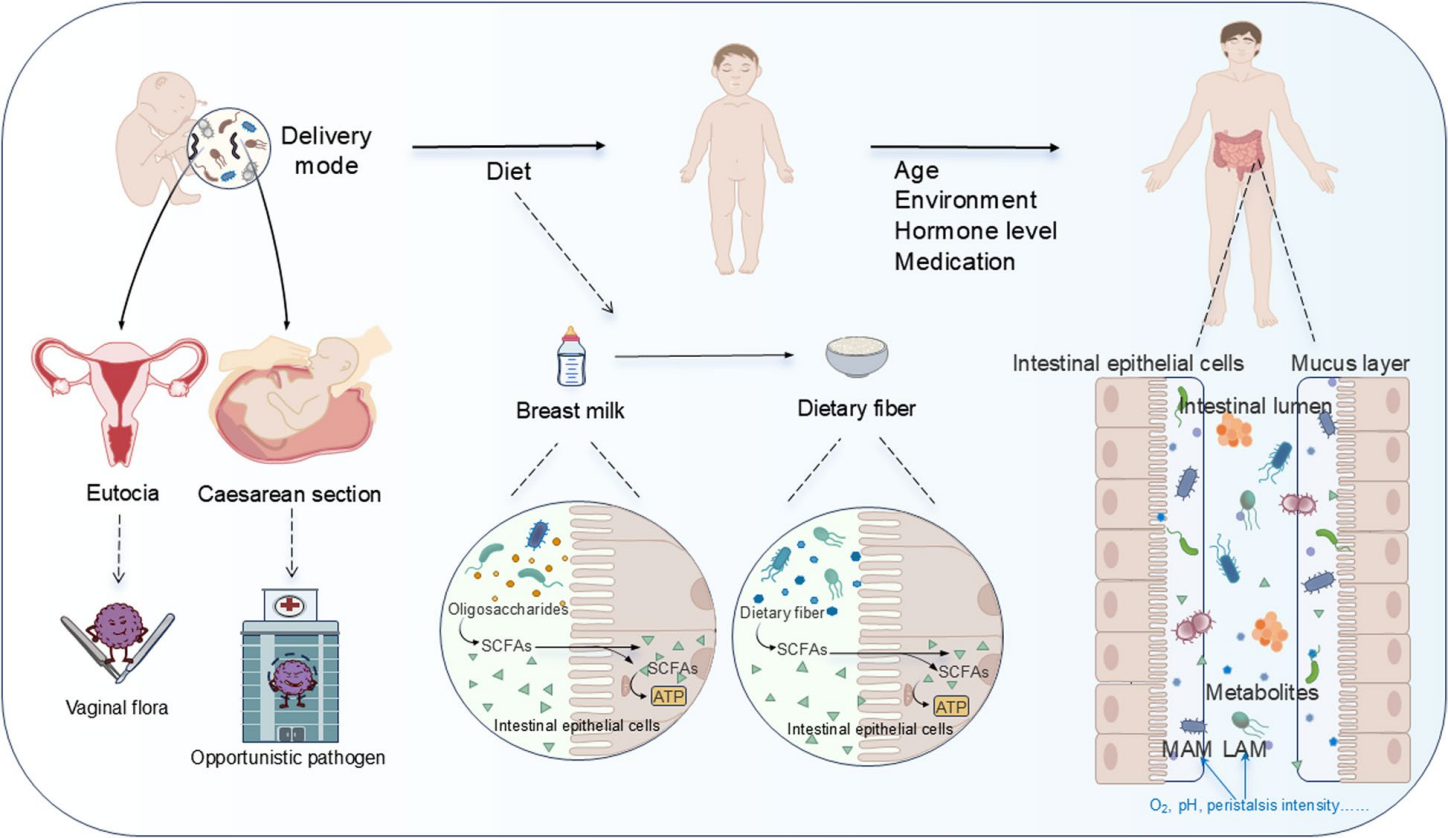
Temporal differences in gut microbiota at the species or genus level. Gut microbiota is transmitted vertically through the mother's body and exposed to the external environment from birth, with a constant value in the gut. In the early stages, different delivery methods significantly affect the composition of GM. Newborns born vaginally are directly inoculated with the mother's feces and vaginal flora during delivery, while newborns born by caesarean section are first exposed to opportunistic pathogens related to the hospital environment; Then, as the dietary structure shifts from liquid breast milk to solid food, the types of bacterial fermentation substrates also undergo significant changes. Therefore, after weaning, the GM of infants undergoes significant changes and gradually transforms into a child-type microbiota. As age increases, the oxygen content in the human intestinal environment continues to decrease, which leads to the gradual replacement of initially colonized aerobic and facultative anaerobic bacteria in the intestine by obligate anaerobic bacteria and tends to stabilize

In terms of temporal differences, GM colonization in the human gut begins at birth through maternal vertical transmission and exposure to the external environment, evolving from a simple and unstable community to a complex and stable one. By approximately three years of age, the composition and distribution of GM resemble those of the adult, maintaining a dynamic equilibrium [11, 12]. Initially, the mode of delivery significantly influences GM composition. Newborns delivered vaginally are exposed to the mother’s vaginal and fecal bacteria, whereas those born via caesarean section encounter opportunistic pathogens associated with the hospital environment, such as *Enterococcus, Enterobacter,* and *Klebsiella.* Studies have shown that the abundance of *Bacteroides* in neonates delivered by caesarean sections is significantly lower compared to those born vaginally [13–15](Fig. 2). Subsequently, *Bifidobacterium,* and *Lactobacillus,* which utilize the oligosaccharides in breast milk, become dominant in the infant gut. As the diet transitions from liquid milk to solid foods, the substrates for bacterial fermentation change markedly, leading to a gradual shift from an infant-type GM to a child-type GM after weaning. During this period, the number of *Bifidobacterium* decreases gradually, while bacteria that degrade plant polysaccharides, such as *Clostridium* increases [16, 17](Fig. 2). Additionally, the oxygen content of the human gut continues to decrease with age, resulting in the gradual replacement of initially colonizing aerobes and facultative aerobes with anaerobic bacteria [14].

In terms of spatial differences, firstly, GM distribution within the human gut exhibits significant heterogeneity, encompassing both horizontal and vertical differences. From the intestinal mucosal surface to the intestinal lumen, differences in physicochemical parameters—such as oxygen concentration, mucus concentration and redox potential—create a horizontal gradient between mucosa-associated microbiota (MAM) and lumen-associated microbiota (LAM) [5, 6](Fig. 2). Furthermore, the human intestine is divided into six anatomical segments: duodenum, jejunum, ileum, cecum, colon and rectum. Physical factors (e.g. intestinal structure, peristaltic intensity and transit time, etc.) and chemical factors (e.g. pH, oxygen content and mucus concentration, etc.) vary among different intestinal segments, forming a distinct vertical gradient [18, 19]. Therefore, the spatial distribution of GM in the human intestine is characterized by a higher abundance and diversity of *Firmicutes* and *Bacteroidetes* in the lower gastrointestinal MAM compared to the upper gastrointestinal MAM, which is dominated by *Proteobacteria* and *Firmicutes* [20, 21]. In the small intestine, the high frequency of peristalsis and the elevated oxygen levels result in a LAM primarily composed of aerobes or facultative aerobes, such as *Proteobacteria* and *Lactobacillales*. In contrast, the LAM of the large intestine predominantly consists of anaerobic bacteria like *Bacteroidales* and *Clostridiales*, owing to slower peristaltic movements and lower concentrations [22]. Secondly, drug therapy can significantly alter the composition of gut microbiota, leading to dysbiosis. For example, antibiotics can cause a sharp decrease in microbial diversity; Proton pump inhibitors (PPIs) can reduce gastric acidity and may lead to overgrowth of small intestinal bacteria; Nonsteroidal anti-inflammatory drugs can cause damage to the intestinal lining, leading to an increase in pathogenic bacteria and a decrease in beneficial bacteria. In addition, diet is one of the most important regulators of gut microbiota composition and function, and different dietary patterns can lead to different microbial characteristics in various parts of the gut. For example, high-fat and high sugar diets are associated with a decrease in microbial diversity and an increase in pro-inflammatory bacteria; A high dietary fiber diet produces short chain fatty acids, which are beneficial for intestinal health; A high protein diet can lead to the production of harmful metabolites such as ammonia and hydrogen sulfide, which may cause a shift towards more proteolytic bacteria, thereby damaging intestinal health.

In summary, the significant differences in the distribution of human GM, especially the vertical variations in its spatial distribution, suggest that GM and its metabolites interact differently with various intestinal segments differently. These interactions influence diverse physiological and pathological processes in humans. Therefore, exploring the relationship between the microbes in specific intestinal segments and diseases will lay the foundation for the development of targeted microbiome-based therapeutics for a range of diseases.

## Physiological functions of different intestinal segments and their corresponding microbiota characteristics

The human intestinal tract, extending from the pylorus of stomach to anus, is a crucial component of the digestive system, It is anatomically and functionally divided into six segments: the duodenum, jejunum, ileum, cecum, colon, and rectum. Each segment exhibits distinct physiological functions and microbial characteristics (Table 1). The small intestine, comprising the duodenum, jejunum and ileum, is enriched with aerobes or facultative anaerobes and is primarily responsible for the digestion and absorption of nutrients. In contrast, the cecum and colon harbor anaerobic bacteria such as *Bacteroides* and *Clostridia*, which contribute to stool formation by fermenting cellulose and absorbing water. Overall, the distinct composition and function of the microbiota in each intestinal segment collaborate to maintain intestinal homeostasis and host health.

**Table 1:** Original microbes in different intestinal segment

Intestinal segment	Function	Microbial content	Main microbes	Refs
Duodenum	Digestion and absorption, acid–base balance	≤ 10^3^ CFU/mL	*Firmicutes, Proteobacteria, Prevotella, Streptococcus, Bacillus, Pseudomonas*	[23–25]
Jejunum	Digestion and absorption	10^3^-10^5^ CFU/mL	*Firmicutes, Proteobacteria, Streptococcus, Prevotella, Veillonella, Escherichia*	[26, 27]
Ileam	Digestion and absorption	10^7^–10^8^ CFU/mL	*Proximal ileum: Micrococcaceae, Streptococcus, Haemophilus, Escherichia* *Terminal ileum: Enterobacteriaceae, Cetobacterium, Cupriavidus, Bacteroides, Escherichia*	[20, 30, 31]
Cecum	Immune defense	10^8^ CFU/mL	*Escherichia coli, Lactobacillus, Enterococcus*	[35, 36]
Colon	Water absorption, feces shaping	10^11^–10^12^ CFU/mL	*Firmicutes, Bacteroidetes, Proteobacteria Verrucomicrobia, Fusobacteria, Desulfobacterota, Actinobacteria, Bacteroides, Faecalibacterium, Escherichia/Shigella, Sutterella, Akkermansia, Parabacteroides, Prevotella, Lachnoclostridium, Alistipes, Fusobacterium, Erysipelatoclostridium, Lachnospiraceae*	[37, 38]
Rectum	Storing and excreting feces	Unknown	*Firmicutes, Bacteroidetes, Proteobacteria, Bacteroidaceae, Lachnospiraceae, Prevotellaceae, Ruminococcaceae, Escherichia, Blautia*	[42–44]

### Duodenal physiological function and microbial characteristics

The duodenum primarily receives digestive juices, including gastric fluid, pancreatic fluid, and bile acid, to facilitate the digestion and absorption of food. It secretes alkaline mucus to maintain acid–base balance and protect the intestinal mucosal barrier. Due to its high oxygen content and rapid food transit speed, the duodenum limits the density and diversity of its microbiota. Consequently, microbial biomass in duodenal aspirates from healthy adults typically does not exceed 10^3^ colony forming units per milliliter (CFU/mL) [23]. Despite its low microbial density and diversity, its unique composition and functional characteristics are closely related to various diseases. By conducting in-depth research on the distribution of duodenal microbiota and its relationship with the host, new ideas can be provided for the diagnosis and treatment of related diseases. In duodenal biopsy samples, *Firmicutes* and *Proteobacteria* predominate, with *Prevotella, Streptococcus, Bacillus,* and *Pseudomonas* being the dominant genera [24, 25]. Research indicates that the duodenal microbiota may be closely associated with the progression of diseases such as functional dyspepsia, celiac disease, pancreatitis, or pancreatic cancer. Further investigation into the duodenal microbiota could provide valuable insights into the development of these diseases and offer potential targets for therapeutic intervention.

### Jejunal physiological function and microbial characteristics

The jejunum, the longest segment of the human intestine with the largest surface area, is rich in various digestive enzymes and serves as the primary site for the digestion and absorption of lipids, glucose, and vitamins. The microbial community density and diversity of the jejunum are between those of the duodenum and ileum, and its composition and functional characteristics are influenced by nutrient supply, oxygen concentration, and bile acids. The microbiota of healthy adults' jejunum comprises approximately 10^3^–10^5^ CFU/mL [26]. Both LAM and MAM are dominated by *Firmicutes* and *Proteobacteria*, with a significant presence of *Streptococcus, Prevotella, Veillonella,* and *Escherichia.* The abundance of other microbial genera varies considerably across different studies [26, 27]. Aerobes or facultative anaerobes such as *Enterococcus, Staphylococcus,* and *Lactobacillus* in jejunum can produce lipase [28, 29]. It is hypothesized that lipase produced by these jejunal microbiota supports the jejunum's role as the main absorption site for dietary lipids. Recent studies suggest that jejunal microbiota may be closely related to conditions such as small intestinal bacterial overgrowth, obesity, and diabetes. Therefore, a deeper understanding of jejunal microbiota could pave the way for novel therapeutic strategies to treat a range of metabolic disorders.

### Ileal physiological function and microbial characteristics

The ileum is uniquely characterized by its roles in digestion, absorption, and microbial composition. It is the primary site for the absorption of amino acids, vitamin C, vitamin B12, bile salts, and so on. The ileal microbiota contains approximately 10^7^–10^8^ CFU/mL. In the proximal ileum, the MAM in the proximal ileum consists of *Micrococcaceae, Streptococcus, Haemophilus,* and *Escherichia* [20, 30], resembling the microbial composition of the upper gastrointestinal tract dominated by *Proteobacteria* and *Firmicutes*. Conversely, the MAM in the distal ileum is dominated by *Enterobacteriaceae, Cetobacterium, Cupriavidus, Bacteroides,* and *Escherichia* [30, 31], similar to the lower gastrointestinal tract's microbiota, which is dominated by *Firmicutes* and *Bacteroidetes*.

The ileal microbiota is involved in lipid emulsification through bile salt hydrolase and hydroxysteroid dehydrogenase, facilitating the enterohepatic circulation and metabolism of bile salts [32, 33]. In addition, the ileal microbiota can promote postprandial glucagon-like peptide-1 (GLP-1) secretion via bile acid-TGR5 signaling, thereby regulating blood glucose levels and appetite [34]. Current research indicates that ileal microbiota may be associated with diseases such as Crohn's disease, colorectal cancer, and circadian rhythm disorders. It is suggested that targeting the ileal microbiota can be used as a new strategy for metabolic-immune disease intervention.

### Cecal physiological function and microbial characteristics

The cecum contains numerous lymph nodes, providing immune defenses against bacterial and viral invasions. Ileocecal valve, a structure within the cecum, prevents the backflow of large intestine contents into the small intestine and regulates the transit of small intestine contents into the large intestine, ensuring adequate digestion and absorption in the small intestine. The cecum's ability to temporarily store excreta creates an ideal environment for the growth and multiplication of microorganisms. Consequently, the cecal microbiota in healthy adults contains approximately 10^8^ CFU/mL, which is about 100 times more than that of the terminal ileum. Predominant microbes in the cecum are facultative anaerobes, including *Escherichia coli, Lactobacillus,* and *Enterococcus*, which account for about 25% of the total caecum anaerobes. In contrast, obligate anaerobes such as *Bacteroides* and *Bifidobacterium* are much less abundant [35, 36]. The human cecum, a blind sac connected to the junction of small intestine and large intestine, presents challenges for biopsy sampling. Additionally, the requirement for endoscopic passage through multiple intestinal segments can lead to microbiota displacement, resulting in unstable compositions in detected cecal microbiota. Therefore, current research on cecal microbiota is primarily limited to a small number of mouse experiments.

### Colonic physiological function and microbial characteristics

The colon, situated between the cecum and the rectum, is part of the large intestine and is divided into four main segments: the ascending colon, transverse colon, descending colon and sigmoid colon. The main functions of the colon include water absorption, vitamin synthesis, stool formation, and facilitation of defecation. In healthy adults, the colonic microbiota content ranges from 10^11^ to 10^12^ CFU/mL [37], predominantly consisting of *Firmicutes* and *Bacteroidetes,* followed by *Proteobacteria, Verrucomicrobia, Fusobacteria, Desulfobacterota,* and *Actinobacteria.* The relative abundance of genera such as *Bacteroides, Faecalibacterium,* and *Escherichia* is notably high [38].

The colonic microbiota exhibits spatial distribution differences. For example, proximal and distal MAM differ in the abundance of *Porphyromonas, Murdochiella, Finegoldia, Anaerococcus,* and *Peptoniphilus*. Similarly, Proximal and distal LAM differ in the abundance of *Bacteroides, Clostridium IV,* and *Oscillibacter* [39, 40]. Therefore, the composition of proximal MAM closely resembles that of proximal LAM, while distal LAM is most similar to that of fecal microbiota. Traditional colon sample collection methods often involve invasive endoscopic techniques, which are prone to bacterial translocation and contamination. To achieve more accurate colon microbiota sampling, new non-invasive technologies, such as smart capsules for targeted proximal colon microbiota sampling, have been developed [41].

### Rectal physiological function and microbial characteristics

The rectum is the final segment of the intestinal tract, connecting the colon to the anus. Its main function is to store and excrete feces. Moreover, the rectal mucosal layer contains numerous glands that secrete mucus, lubricating the rectum and reducing friction during fecal passage. Comparative analyses of microbiota in feces, rectal swabs, and rectal mucosa of adults reveal that the dominant microbes across all three sample types are *Firmicutes, Bacteroidetes,* and *Proteobacteria*. Notably, *Blautia* (from *Firmicutes*) is enriched in fecal samples, while *Bacillus* (from *Proteobacteria*) is enriched in rectal swab samples. However, the *α-diversity* of rectal MAM is significantly lower, with predominant microbes including *Bacteroidaceae, Ruminococcaceae, Escherichia,* and *Blautia* [42–44]. Furthermore, differences in the functional pathways of microbiota between fecal and rectal swab samples are smaller than those between fecal and rectal mucosa samples. Specifically, MAM are relatively enriched for pathways related to glycolysis and the biosynthesis of nucleosides, adenosine, guanosine and inosine. In contrast, microbiota in stool and rectal swab samples differ in the pathways related to peptidoglycan, galactose, stachyose, purine and pyrimidine metabolism [44]. In conclusion, rectal swabs are considered a promising alternative to stool sampling for gut microbiota analysis.

In summary, each intestinal segment possesses unique physiological functions and distinct microbiota compositions that contribute to overall gut homeostasis and host health. Understanding the specific interactions between microbiota and their respective intestinal environments is essential for elucidating their roles in various physiological and pathological processes. This knowledge lays the groundwork for developing targeted microbiome-based diagnostic and therapeutic strategies for a wide range of diseases.

## Effects of gut microbiota in different segments on disease progression

GM is intricately linked to numerous physiological and pathological processes in humans. Alterations in its composition and distribution significantly influence the progression of various clinical diseases. However, most research has primarily utilized fecal samples to explore the relationship between diseases and GM, with limited focus on potential differences in microbiota across different anatomical sites [45]. Recent studies have revealed substantial disparities between the microbiota and its metabolites in the actual gut environment compared to those in fecal samples, indicating that fecal microbiota and metabolites are not fully representative of the microbial composition and metabolic status of the entire intestine [46, 47]. The composition of the microbiota varies across different intestinal segments, including duodenum, jejunum, ileum, cecum, colon and rectum, and their variations exert distinct effects on disease procession (Table 2). Recently, some studies have begun exploring the relationship between diseases and microbial changes in specific intestinal segments, which could facilitate the precise regulation of the microbiota in targeted intestinal segments for microbiome-based therapeutics in related diseases.

**Table 2:** Alterations of microbes in different intestinal segment affecting different diseases and their potential mechanisms

Intestinal segment	Subject	Diseases	Altered microbes	Possible effects	Refs.
Duodenum	Human	Functional dyspepsia	Upregulated: *Firmicutes*, *Rothia*, *Clostridium*, *Haemophilus*, *Actinobacillus* Downregulated: *Bacteroidetes*, *Fusobacteria*	The relative abundances of *Streptococcus*, *Prevotella* and *Veillonella spp* was negatively correlated with gastric emptying time	[49–51]
	Human	Celiac disease	Upregulated: *Proteobacteria*, *Enterobacter*, *Streptococcus*, *Escherichia* Downregulated: *Firmicutes*, *Bacteroidetes*, *Akkermansia*, *Clostridium, Bacillus*	The abundance of *Neisseria* is related to the degree of intestinal villus atrophy	[54–56]
	Human	Acute pancreatitis	Upregulated: *Firmicutes, Microtrichales, Pseudomonas, Ruminococcaceae, Pediococcus* Downregulated: *Proteobacteria, Bacteroidetes, Bacteria, Prevotellaceae, Bilophila, Candidatus Saccharimonas, Treponema, Stomatobaculum*	Unknown	[59]
	Human	Pancreatic cancer	Upregulated: *Acinetobacter, Aquabacterium, Oceanobacillus, Rahnella, Massilia, Delftia*, *Deinococcus, Sphingobium* Downregulated: *Porphyromonas, Paenibacillus, Enhydrobacter, Escherichia, Shigella, Pseudomonas*	Unknown	[60, 61]
Jejunum	Human	Small intestinal bacteria overgrowth	Main: *Klebsiella, Enterobacter, Enterococcus faecalis, Escherichia coli, Pseudomonas aeruginosa, Staphylococcus aureus*	*Lactobacillus* translocation growth in the jejunum will lead to poor fat absorption	[65]
	Human	Morbidly obese with high insulin resistance	Upregulated: *Bacteroides, Streptococcus, Alkalibacterium*	Unknown	[68]
	Wistar rats	Type 2 diabetes mellitus	Upregulated: *Firmicutes, Staphylococcus sciuri* Downregulated: *Proteobacteria, Bacteroidetes, Acidobacteria, TM7, Gemmatimonadetes, Shigella, Alcaligenes, Bacteroides, Clostridiaceae Clostridium, Allobaculum*	Intestinal permeability was significantly negatively correlated with *Bacteroides fragilis*	[70, 71]
Ileam	Human	Crohn's disease	Upregulated: *Bacteroides* (active stage), *Streptococcus*, *Veillonella*, and *Lactococcus* (remission stage)	Unknown	[74]
	Human	Proximal colon cancer	Upregulated: *Bacteroides fragilis* (better prognosis), *Prevotella* (worse prognosis)	Ileal apoptosis correlated with the relative abundance of *Erysipelotrichaceae*, but negatively correlated with that of *Fusobacteriaceae* families The overrepresentation of *Fusobacteriaceae* family members in the ilea was also negatively correlated with TIL abundance	[78]
	CDKO mice	Circadian disruptions	Upregulated: *Erysipelotrichaceae, Lactobacillaceae*	Unknown	[81]
Cecum	Balb/c mice	Liver injury induced by furan	Upregulated: *Cyanobacteria, Rhodospirillaceae, Olsenella, Sporobacter, Anaerovorax, Insolitispirillum, Blautia* Downregulated: *Eisenbergiella*	*Cyanobacteria* produce *Microcystins-LR*, which can disorder liver actions and deteriorate redox balance	[85, 86]
	C57BL/6 J mice	Nonalcoholic fatty liver disease	Upregulated: *Firmicutes* Downregulated: *Bacteroidetes*	*Veillonella* and *Olsenella* were positively correlated with the serum levels of ALT and ALP, respectively *Paraprevotella*, *Clostridium XI*, and *Barnesiella* were negatively correlated with HDL-C accumulation, while *Eubacterium, Akkermansia, Alloprevotella*, and *Parabacteroides* were positively related to HDL-C content	[87]
	Wistar rats	Hyperlipidemia	Upregulated: *Firmicutes, Turicibacter, Bifidobacterium* Downregulated: *Euryarchaeota, Actinobacteria, Tenericutes, Saccharibacteria, Corynebacterium_1, Nosocomiicoccus, Jeotgalicoccus*	*Firmicutes* could absorb the calories in the diet and increase the fat storage in the body *Turicibacter* had a strong positive correction with body weight gain and energy harvest	[88]
	Human	Irritable bowel syndrome (diarrhoea-predominant)	Upregulated: *Ruminococcus torques* Downregulated: *Raoultella, Fusobacterium*	*Ruminococcustor* *torques* was positively associated with HAM-A, HAM-D, EC cell number, IBS-SSS, degree of abdominal pain, frequency of abdominal pain and frequency of defecation	[89–91]
Colon	Human	Inflammatory bowel disease	Upregulated: *Enterobacteriaceae* Downregulated: *Bacteroides, Parabacteroides distasonis, Rikenellaceae, Coprococcus, Lachnospiraceae, Faecalibacterium prausnitzii, Ruminococcaceae*	The decrease of butyrate-producing bacteria reduces the concentration of butyrate, which affects the energy metabolism and inflammatory response of colon cells	[94, 95]
	Human	Irritable bowel syndrome	Upregulated: *Prevotella copri, Eubacterium dolichum, Veillonella dispar, Haemophilus parainfluenzae* Downregulated: *Anaerostipes*	*Prevotella* *copri* has been associated to enhancing susceptibility to inflammatory disorders through intrinsic Th17 promoting capability, driving cytokines IL-6 and IL-23	[94, 97]
	Human	Colorectal cancer	Upregulated: *Fusobacterium nucleatum, Bacteroides fragilis*	The FadA adhesin expressed by *Fusobacterium nucleatum* binds to E-cadherin that activates β-catenin, promotes the expression of oncogenes and transcription factors, and leads to the growth of colorectal cancer cells *Enterotoxin* produced by *Bacteroides* *fragilis* can induce the activation of activator of transcription 3 to drive Th17-type immune response	[98–101]
	Human	Parkinson's desease	Upregulated: *Ralstonia* Downregulated: *Faecalibacterium*	The abundance of butyrate-producing bacteria was significantly lower	[103, 104]
	CD1 mice	Sleep deprivation	Upregulated: *Aeromonas* Downregulated: *Akkermansia, Bacteroides, Faecalibacterium*	Unknown	[107]
	Human	Oral diseases	Upregulated:*Bacteroides, Proteobacteria* Downregulated: *Faecalibacterium*	The relative abundance of *Faecalibacterium* was lower with more tooth loss	[110]
	Human	Calcium oxalate urolithiasis	Downregulated: *Oxalobacter formigenes*	*Oxalobacter formigenes* degrades oxalates and transports them through the intestinal epithelium	[111–113]
	Human	Minimal hepatic encephalopathy	Downregulated: *Clostridiaceae, Bacteroides*	Unknown	[114, 115]
Rectum	Human	Infertility	Upregulated: *Firmicutes, Hungatella, Dorea, Peptoniphilus* Downregulated: *Bacteroidetes, Bacteroides, Prevotella 9, Ruminococcaceae UCG-004, Ruminococcaceae UCG-010*	*Prevotella* can produce short-chain fatty acids to construct intestinal mucosal barrier *Bacteroides* can prevent intestinal mucus synthesis by producing metabolites	[116, 117]
	Human	Alcohol-related cirrhosis	Downregulated: *Escherichia coli, Enterobacteriaceae*	Unknown	[121]
	Human	Ulcerative colitis	Upregulated: *Bacteroidetes, Blautia, Ruminoccocaceae, Lachnospiraceae* Downregulated: *Proteobacteria, Bacteroides, Prevotella*	Unknown	[122]
	Human	Acute pancreatitis (AP)	Upregulated: *Finegoldia* (mild AP)*, Anaerococcus* (moderately severe AP)*, Enterococcus* (severe AP) Downregulated: *Blautia* (mild AP)*, Eubacterium hallii* (moderately severe AP and severe AP)	Unknown	[123]

### Duodenal microbiota and disease

Functional dyspepsia (FD) is a clinical syndrome characterized by epigastric pain or burning, postprandial fullness, or early satiety lasting for at least 6 months [48]. Studies have shown that the duodenal microbiota of FD patients undergoes significant changes in microbial biomass and diversity. For example, Shanahan [49] found that the relative abundance of *Firmicutes, Bacteroidetes* and *Fusobacteria* in the duodenal MAM of 56 FD patients correlated with the severity of disease symptoms. At the genus level, the relative abundance of *Rothia, Clostridium, Haemophilus,* and *Actinobacillus* significantly increased [50], while the abundance of *Streptococcus, Prevotella,* and *Veillonella spp*. was negatively correlated with gastric emptying time [51]. Additionally, the duodenal mucosa of FD patients exhibits low-grade inflammation and destruction of the mucosal barrier, with increased mucosal permeability correlating with the severity of inflammation, but whether the microbiota is involved in this process is not clear [52]. These findings suggest that duodenal inflammation in FD patients may be driven by dysbiosis, although the exact pathogenesis—such as how duodenal microbiota contributes to intestinal inflammation and gastrointestinal symptoms—requires further investigation.

Celiac disease (CeD) is an inflammatory disease of the small intestinal mucosa triggered by intolerance to gluten containing cereals [53]. In CeD patients, the duodenal microbiota shows an increase in *Proteobacteria* and a decrease in *Firmicutes* and *Bacteroidetes*. At the genus level, there is an increase in *Enterobacter, Streptococcus* and *Escherichia,* alongside a decrease in *Akkermansia, Clostridium,* and *Bacillus* [54–56]. Moreover, the abundance of *Neisseria* correlates with the degree of intestinal villus atrophy. Notably, *Neisseria flavescens* can evade lysosomal degradation in Caco-2 cells and induce inflammatory responses in dendritic cells and isolated mucosal explants [57]. In addition, *Pseudomonas* *aeruginosa* participates in gluten hydrolysis, altering its immunogenicity and activating the innate immune pathway associated with CeD [58]. These findings indicate that the duodenal microbiota may stimulate the intestinal immune system and induce a variety of inflammatory signals in CeD by degrading gluten into immunogenic peptides. However, the specific mechanism remains to be fully elucidated and verified.

Recent research has identified the impact of duodenal microbiota on the pathogenesis of pancreatitis and pancreatic cancer. In the duodenal MAM of patients with acute pancreatitis (AP), there is a significant increase in the relative abundance of *Pseudomonas, Ruminococcaceae,* and *Pediococcus*, along with enriched functional pathway related to the endocrine system, glycerolipid metabolism, and dioxin degradation [59]. Additionally, in patients with pancreatic cancer, the duodenal MAM shows higher relative abundances of *Acinetobacter, Aquabacterium, Oceanobacillus, Rahnella, Massilia, Delftia, Deinococcus,* and *Sphingobium* were higher, while *Bacteroides* abundance decreases, possibly linked to increased duodenal mucosal inflammation [60, 61]. Kohi [62] also found that in patients with pancreatic ductal adenocarcinoma (PDAC), the duodenal microbiota was dominated by *Enterococcaceae, Lactobacillaceae,* and *Bifidobacteriaceae* dominated the duodenal microbiota, and *Fusobacterium, Rothia,* and *Neisseria* significantly enriched in short-term PDAC survivors. These findings suggest that alterations in the abundance of specific duodenal microbiota may induce pancreatic inflammation and affect pancreatic cancer progression by modulating related human metabolic pathways. Consequently, the role of duodenal microbiota in pancreatic cancer needs to be further investigated to clarify its potential in better monitoring patients for pancreatic cancer risk.

### Jejunal microbiota and disease

Small intestinal bacterial overgrowth (SIBO) has traditionally been diagnosed using small bowel aspirate, considered the ‘gold standard’, with a threshold of 10^5^ CFU/mL in jejunal aspirate and 10^3^ CFU/mL in duodenal aspirate. However, glucose and lactulose breath tests are now commonly used in clinical practice for SIBO diagnosis: defined by an increase in H_2_ concentration of 20 parts per million (ppm) from baseline within 90 min and an increase in CH_4_ levels ≥ 10 ppm at any time [63]. Common clinical symptoms of SIBO include abdominal pain, bloating, diarrhea, flatulence and dyspepsia [64], which may be associated with translocation and overgrowth of jejunal microbiota. For example, Pistiki [65] isolated approximately 170 species of aerobes from the jejunum of 117 SIBO patients, mainly including *Klebsiella, Enterobacter, Enterococcus faecalis, Escherichia coli, Pseudomonas aeruginosa,* and *Staphylococcus aureu*s. Additionally, small bowel surgery, radiation or radiotherapy can all lead to the occurrence of bacterial translocation [66], due to factors such as inhibited intestinal peristalsis, abnormal intestinal structure or reduced gastric acid secretion, providing opportunities for bacterial overgrowth. This overgrowth results in the excessive production of acid and gas, causing abdominal pain and bloating [67]. Furthermore, the growth of certain translocated microbiota in the jejunum can lead to metabolic disorders. For instance, *Lactobacillus* can produce bile salt hydrolase or lipase, leading to the depolymerization and premature reabsorption of conjugated bile acids in the jejunum instead of the ileum. This interference with enterohepatic circulation results in fat malabsorption [28, 29]. Meanwhile, free bile acids, which are toxic to the intestinal mucosa, can lead to bile acid diarrhea and further malabsorption [66]. In summary, the translocation and overgrowth of jejunal microbiota are closely related to the development of SIBO, and changes in the abundance of different strains trigger various gastrointestinal symptoms. However, the specific roles of many strains in the jejunal microbiota in SIBO require further studies.

The jejunal microbiota has also been implicated in diseases related to dysregulation of glucose homeostasis, such as obesity or diabetes. Carolina [68] observed that in the jejunal MAM of morbidly obese patients with high insulin resistance, the abundance of *Proteobacteria, Fusobacteria,* and *Bacteroidetes* significantly increased. Metformin treatment notably upregulated the relative abundance of *Halomonadaceae* and markedly slowed disease progression, suggesting that *Halomonadaceae* in the jejunal microbiota plays a role in morbidly obese patients with high insulin resistance. In addition, Jayashree [69] found that intestinal permeability (IP) was significantly higher in patients with type 2 diabetes mellitus (T2DM) compared to healthy adults, a finding also observed in T2DM rat models compared to normal rats [70]. The level of IP was significantly negatively correlated with the relative abundance of *Bacteroides fragilis* [71], indicating that a decrease in *Bacteroides fragilis* may be a critical factor contributing to jejunal barrier damage. Furthermore, the jejunal microbiota of the T2DM rat model exhibited a significant increase in *Proteobacteria, Bacteroidetes, Acidobacteria, Gemmatimonadetes,* and *TM7.* At the genus level, *Shigella, Alcaligenes, Bacteroides, Allobaculum,* and *Clostridiaceae Clostridium* were also significantly reduced [71]. Current research suggests that glucose degradation may be the main pathway through which the jejunal microbiota regulates host metabolism to influence obesity and T2DM [72]. In general, disturbances in the jejunal microbiota may lead to damage of the jejunal barrier, contributing to the pathogenesis of obesity or diabetes. However, the association between jejunal microbiota and glucose metabolism requires further exploration and clarification.

In summary, each intestinal segment harbors unique physiological functions and distinct microbiota compositions that collectively contribute to overall gut homeostasis and host health. Understanding the specific interactions between microbiota and their respective intestinal environments is essential for elucidating their roles in various physiological and pathological processes. This knowledge lays the foundation for developing targeted microbiome-based diagnostic and therapeutic strategies for a wide range of diseases.

### Ileal microbiota and disease

Crohn's disease (CD) is a chronic inflammatory bowel disease with an unclear etiology. Studies have demonstrated significant differences in the composition of the ileal microbiota across various stages of CD, suggesting that interactions between the ileal microbiota and the host play a crucial role in the disease's pathogenesis [73]. For example, the relative abundance of *Bacteroides* is higher during the active stage of CD, whereas *Streptococcus, Veillonella,* and *Lactococcus* were higher in the remission. These bacteria are primarily involved in amino acid and carbohydrate transport and metabolism [74]. Yael [75] found that specific ileal microbiota of CD patients are associated with the co-expression genes APOA1 and DUOX2 in the ileum. Specifically, up-regulation of DUOX2 gene correlates with an increase in *Proteobacteria,* while downregulation of APOA1 is associated with a decrease in *Firmicutes* and *Bacteroidetes*. These changes promote oxidative stress and Th1 cell polarisation, exacerbating intestinal mucosal damage in CD patients. Additionally, recurrence of CD following ileal resection is common and may be related to a reduction in the alpha diversity of the ileal mucosa-associated microbiota (MAM), an increase in certain *Proteobacteria* members, and a decrease in members of the *Lachnospiraceae* and the *Ruminococcaceae* families within the *Firmicutes* phylum [76, 77]. These microbial alterations may serve as more specific predictors of CD recurrence risk compared to general clinical factors. Overall, the distinct microbial and genetic profiles of the ileum identify it as a primary inductive site for all forms of CD, potentially aiding in the diagnosis of different disease stages, therapeutic selection, and prognosis evaluation.

The ileal microbiota also influences the local immune microenvironment and participates in immune surveillance of colon cancer (CC). Research indicates that ileal microbiota affects the therapeutic efficacy of oxaliplatin in CC patients by modulating tumor immunosurveillance. Marion [78] observed that *Bacteroides fragilis* was the only enriched ileal microbe in CC patients with a favorable prognosis, whereas *Paraprevotella clara* predominated in those with a poor prognosis. In the CC mouse model, mice supplemented with *Bacteroides fragilis* exhibited higher levels of tumor-infiltrating lymphocytes (TILs) and lower levels of CD45 cells in the ileum compared to those supplemented with *Paraprevotella clara* [78]. These findings align with previous studies showing that* B. fragilis* induces dendritic cells to release IL-1β and IL-12, increasing the number of follicular helper T (Tfh) cells, while *Fusobacterium nucleatum* promotes Th17 cell accumulation in tumor-draining lymph nodes, enhancing the immune response [79, 80]. Thus, specific ileal microbiota, such as *B. fragilis* and *F. nucleatum*, may be involved in the pathogenesis and therapeutic response of CC by modulating the ileal immune response.

Recent studies have also linked the ileal microbiota to the host's circadian rhythm. For example, Ana Carolina [81] found that in Cry1^−/−^Cry2^−/−^rhythm gene knockout mouse model, eating and sleeping rhythms were significantly disrupted, leading to the complete loss of microbial cycling in the ileum Additionally, *Erysipelotrichaceae* and *Lactobacillaceae* were significantly increased in the ileal microbiota. This disruption mirrors findings from other studies where knockout of clock genes such as Bmal and Per led to disturbances in the microbial circadian rhythm [82]. Furthermore, the ileal microbiota appears to be linked to the circadian rhythm of the host liver. Disruption of the microbiota's circadian dynamics affects the liver transcriptome, leading to non-rhythmic expression of genes involved in chromatin and nucleosome assembly, as well as the metabolism of amino acids, polysaccharides, lipids, and steroids [83, 84]. Consequently, the host's molecular biological clock is intertwined with the diurnal dynamics of the ileal microbiota. Interference with the host's biological clock may disrupt the circadian rhythm of the ileal microbiota, subsequently affecting hepatic metabolic rhythms.

### Cecal microbiota and disease

The cecal microbiota is associated with diseases related to abnormal lipid metabolism. For example, Yuan [85] found a significant increase in the relative abundance of *Cyanobacteria, Rhodospirillaceae, Olsenella, Sporobacter, Anaerovorax, Insolitispirillum,* and *Blautia* in the cecal microbiota of mice with furan-induced liver injury. Notably,* Cyanobacteria* produce Microcystins-LR, which can disrupt redox balance and impair liver function in mice [86]. Therefore, *Cyanobacteria* may serve as a potential marker for liver injury, providing new ideas for subsequent research on liver injury. Additionally, Xueliang [87] found that in the cecal microbiota of mice with non-alcoholic fatty liver disease(NAFLD), the relative abundance of *Bacteroidetes* was low, while *Firmicutes* was high, resulting in an increased *Firmicutes / Bacteroidetes* ratio. Microbes positively correlated with lipid content, such as *Paraprevotella, Clostridium Xl,* and *Barnesiella,* were increased, whereas those negatively correlated with fat accumulation, such as *Akkermansia, Alloprevotella,* and *Parabacteroides* were decreased. Additionally, *Veillonella* and *Olsonella* were positively correlated with serum ALT and ALP levels [87]. In rat models of high-fat diet (HFD)-induced hyperlipidemia, there was an increase in *Firmicutes* and a decrease in *Euryarchaeota, Actinobacteria,* and *Tenericutes* within the cecal microbiota [88]. Furthermore, there was also a significant decrease in *Corynebacterium_1*, *Nosocomiicoccus,* and *Jeotgalicoccus,* alongside an increase in Turicibacter and *Bifidobacterium.* Metabolites such as linoleic acid and sphingosine were also significantly elevated [88]. To sum up, the cecal microbiota may play a role in regulating diseases like hyperlipidemia and NAFLD through disruptions in lipid metabolism. However, the specific mechanisms remain to be further explored.

The Cecal microbiota is also implicated in other diseases. For example, in the cecal samples from patients with diarrhea-predominant irritable bowel syndrome (IBS-D), the relative abundance of *Ruminococcus torques* increased, and the relative abundance of *Ruminococcus torques* decreased [89]. This aligns with studies showing a positive correlation between *Ruminococcus torques* and enterochromaffin cells, IBS-SSS, degree of abdominal pain, frequency of abdominal pain, and frequency of defecation [90, 91]. Additionally, a significant decrease in short-chain fatty acids that enhance macrophage function to control inflammation, was found in the cecal contents of mice with Klebsiella pneumoniae-Induced Pneumosepsis [92]. This suggests that bacterial pneumonia can dramatically alter the GM and metabolites, leading to a dysregulated immune response to the lung infections. In conclusion, while the cecal microbiota has been preliminarily linked to various diseases, the specific roles and mechanisms require further investigation, including validation in clinical translational studies.

### Colonic microbiota and disease

The colonic microbiota is relevant with several diseases, such as inflammatory bowel disease (IBD), irritable bowel syndrome (IBS), and colorectal cancer (CRC). IBD is a chronic and recurrent inflammatory disease of the digestive system mediated by immune response, including CD and ulcerative colitis (UC) [93]. In IBD patients, there is a significant increase in *Enterobacteriaceae* and a notable decrease in butyrate-producing microbes such as *Faecalibacterium prausnitzii* and members of the *Ruminococcaceae* family. This results in lower butyrate concentrations, negatively impacting energy metabolism and inflammatory responses in colon cells [94, 95].

IBS is a functional gastrointestinal disorder with symptoms that include abdominal pain and changes in stool or frequency [96]. In the colonic MAM of IBS patients, there is a significant increase in *Prevotella copri, Eubacterium dolichum, Veillonella dispar,* and *Haemophilus parainfluenzae,* along with a significant decrease in *Anaerostipes* [94]. Notably, *Prevotella copri* may be associated with susceptibility to inflammatory diseases by promoting the activation of Th17 cells and driving cytokines IL-6 and IL-23 [97]. Regarding CRC, the colonic microbiota has been extensively studied. *Fusobacterium nucleatum* in humans and mouse models produces the FadA adhesin binding to E-cadherin, which can activate the β-catenin pathway and promote the expression of transcription factors and oncogenes, thereby facilitating the growth of CRC cells [98–100]. Additionally, enterotoxin produced by *B. fragilis* induces the activation of activating transcription factor 3 to drive a Th17-type cellular immune response, which promotes inflammatory responses in the colonic mucosa in CRC by IL-23-driven production of IL-17A [101]. Therefore, *F. nucleatum* and *B. fragilis* are being explored as potential biomarkers for the diagnosis and treatment of CRC. Moreover, butyrate, a metabolite produced by the colonic microbiota, can induce G1 phase arrest or extensive apoptosis in CRC cells, which is an adjunct to conventional chemotherapy for CRC [102]. These findings suggest that further study of specific colonic microbiota will facilitate the development of new strategies for the identification, diagnosis, or treatment of IBD, IBS, and CRC.

*Faecalibacterium,* a key genus within the colonic microbiota, is associated with colitis—a complication of various diseases. For example, in the colonic MAM of Parkinson's disease (PD) patients, the relative abundance of *Faecalibacterium* (anti-inflammatory, butyrate-producing microbe) is significantly reduced, while *Ralstonia *(pro-inflammatory Proteobacteria) is increased. This imbalance may contribute to intestinal inflammation, α-synuclein misfolding, and subsequent neuroinflammation in PD [103, 104]. These observations are consistent with studies indicating that PD patients often exhibit colonic inflammation, which affects neuroinflammation via the brain-gut axis [105, 106]. In another study, Lou Ming [107] found in sleep-deprived mouse models, there was a decrease in the diversity and abundance of colonic microbiota, especially probiotics such as *Akkermansia, Bacteroides,* and *Faecalibacterium,* alongside a significant increase in pathogens like *Aeromonas*. This microbial shift may contribute to colonic mucosal damage. Interestingly, melatonin treatment upregulated *Faecalibacterium* abundance and its metabolite butyrate, thereby preventing colitis induced by acute sleep deprivation in mice [108, 109]. In addition, Anthony [110] demonstrated that changes in the anti-inflammatory status mediated by *Faecalibacterium* in the colonic microbiota may be a key factor in poor oral health. Individuals with more tooth loss exhibited a lower relative abundance of *Faecalibacterium* and a higher relative abundance of *Proteobacteria* in the colonic MAM [110]. These findings highlight the critical role of *Faecalibacterium* in the colonic microbiota in the development of various colitis-associated diseases, including PD, sleep deprivation-related conditions, and oral diseases.

Disturbance in the colonic microbiota can also lead to the formation of calcium oxalate kidney stones and hepatic encephalopathy. For example, *Oxalobacter formigenes*, a member of the colonic microbiota, degrades oxalate to obtain energy, thereby reducing intestinal oxalate secretion and urinary oxalate excretion. This makes it a potential as probiotics for the treatment of hyperoxaluria and calcium oxalate kidney stones [111–113]. Additionally, a decrease in diversity of the colonic MAM and significantly lower numbers of *Bacteroides* contribute to the development of mild hepatic encephalopathy in cirrhotic patients through gut-liver-brain axis [114, 115]. These findings confirm that the colonic microbiota can modulate the progression of various diseases through multiple gut-organ axes.

In summary, each intestinal segment possesses unique physiological functions and distinct microbiota compositions that collectively contribute to overall gut homeostasis and host health. Understanding the specific interactions between microbiota and their respective intestinal environments is essential for elucidating their roles in various physiological and pathological processes. This knowledge lays the foundation for developing targeted microbiome-based diagnostic and therapeutic strategies for a wide range of diseases.

### Rectal microbiota and disease

Alterations in the rectal microbiota have been implicated in various gynecological and systemic diseases. For example, Azpiroz [116] observed a significant reduction in rectal microbiota diversity in infertile women. Specifically, there was an increased *Firmicutes/Bacteroidetes* ratio and a decreased relative abundance of *Prevotella,* which is essential for producing short-chain fatty acids (SCFAs) that help construct the intestinal mucosal barrier. Concurrently, an increase in *Bacteroides*, which can prevent intestinal mucus synthesis by producing metabolites, was noted. These changes collectively weaken the rectal mucosal barrier in infertile women [117]. Additionally, infertile women experiencing repeated implantation failure exhibited an elevated presence of *Erysipelotrichaceae* in their rectal microbiota. This bacterial family induces TNF-α-driven inflammation and insulin-resistant obesity, ultimately contributing to implantation failure [117].

The anatomical proximity between the female urogenital tract and the anorectal region facilitates microbial translocation, potentially linking rectal microbiota alterations to female infertility [118]. Supporting this, the presence of *Prevotella* in both rectum and genital tract has been associated with an increased risk of genital inflammation and HIV infection [119]. These findings suggest that the interaction between rectal microbiota and urogenital microbiota may become a focal point for future research in gynecological diseases.

Beyond gynecological conditions, rectal microbiota is connected to other health issues. In patients with community-acquired pneumonia (CAP) hospitalized for one month, rectal swab analysis revealed that changes in the rectal microbiota influenced cytokine levels and degranulated products. Specifically, alterations in the abundance of butyrate-producing bacteria were associated with changes in IL-27 and IFN-ɣ expression, potentially leading to recurrent infections and rehospitalization post-CAP recovery [120]. Furthermore, David [121] utilized rectal swabs to examine the rectal microbiota of patients with alcohol-associated cirrhosis, finding a significantly lower abundance of *Enterobacteriaceae,* particularly *Escherichia coli*, compared to non-alcohol-associated cirrhosis. This suggests that *Enterobacteriaceae* characteristics could differentiate cirrhosis based on etiologies.

Yu-Fei [122] conducted rectal biopsies in patients with subclinical ulcerative colitis (UC) and identified an increase in *Bacteroidetes, Blautia, Ruminoccocaceae,* and *Lachnospiraceae,* alongside a decrease in *Proteobacteria, Bacteroides, Prevotella,* and *Faecalibacterium* in their rectal MAM. These microbial patterns may serve as biomarkers to distinguish subclinical UC from healthy individuals. Additionally, Shanshan [123] discovered that in patients with acute pancreatitis (AP), the rectal microbiota exhibited elevated levels of *Finegoldia, Eubacterium_hallii,* and *Lachnospiraceae,* which could serve as diagnostic biomarkers for mild AP, while *Eubacterium_hallii* and *Anaerococcus* were indicative of moderately severe AP.

In conclusion, the rectal microbiota plays a significant role in various diseases, interacting with multiple human organs. These insights provide a valuable foundation for future research into the mechanisms underlying related diseases and highlight the potential of rectal microbiota as diagnostic and therapeutic targets.

### Microbiota in different intestinal segments in the same disease

The microbiota composition varies across different intestinal segments, and these variations can influence disease progression and treatment outcomes. Examining multiple segments within the same disease context provides a comprehensive understanding of microbiota-related pathophysiological mechanisms.

Small Intestinal Bacterial Overgrowth (SIBO): According to the American College of Gastroenterology (ACG) clinical guidelines, SIBO is diagnosed when bacterial colony count exceeds 10^3^ CFU/mL in duodenal aspirates or 10^5^ CFU/mL in proximal jejunal aspirates [23]. In SIBO patients, the duodenal microbiota shows a significant increase in *Escherichia* and *Klebsiella*, which correlates with symptom severity and adverse effects on metabolic pathways [124]. Specifically, *Escherichia* is associated with abdominal distension and diarrhea, whereas *Klebsiella* relates to abdominal pain through histamine-induced immune responses that heighten visceral neuron sensitivity, exacerbating pain [125]. Additionally, SIBO patients exhibit enhanced sugar degradation and fermentation pathways leading to increased H_2_ production, supporting the validity of breath tests for SIBO diagnosis [124]. These findings align with Pistiki's research, which identified *E. coli*, *Klebsiella, Enterobacter,* and *Enterococcus* faecalis as predominant pathogenic bacteria in jejunal microbiota of SIBO patients [65]. This suggests that *Escherichia* and *Klebsiella* are the key pathogens in both duodenum and jejunum, underscoring the importance of targeted microbiome-based therapies for SIBO.

Irritable Bowel Syndrome (IBS): While most studies on IBS have focused on colonic microbiota dysbiosis, recent research highlights alterations in other intestinal segments as well. In IBS-D (diarrhea-predominant) patients, an increased abundance of *Bacillus* was observed in the duodenal microbiota, alongside a significant enrichment of *Bacteroides* and a reduction in *Faecalibacterium* in the rectal MAM [126, 127]. *Faecalibacterium* is known to enhance intestinal immunity and maintain the mucosal barrier through anti-inflammatory protein production [128]. Furthermore, Zhu [126] further identified site-specific microorganisms, including *Bacteroides, Prevotella,* and *Oscillospira,* in the rectal MAM of IBS-D patients using LEfSe analysis. A diagnostic model based on these microorganisms demonstrated reliable clinical identification of IBS-D. These results indicate that rectal MAM may have greater diagnostic significance for IBS-D compared to duodenal microbiota, highlighting their potential in IBS-D diagnosis and treatment.

Crohn's Disease (CD): CD primarily affects the terminal ileum but can extend throughout the digestive tract in a segmental distribution, potentially linked to microbiota changes across different segments. In CD patients, the gut microbiota composition varies between the jejunum, ileum, colon, and rectum. For example, both the jejunal and ileal microbiota of CD patients show an increased abundance of *E. coli* and *B. fragilis,* while the colonic microbiota exhibits elevated levels of *E. coli* and *Lactobacillus* [129]. Additionally, *E. coli* is significantly more abundant in the rectal MAM [130]. These findings suggest that adherent and invasive *E. coli* play an important role in the process of CD. Various targeted approaches, such as FimH antagonists, probiotics and phages, have been employed to prevent and treat CD by targeting *E. coli* [131, 132]. The widespread presence of *E. coli* across multiple intestinal segments may contribute to the "leapfrog" distribution of CrD lesions. However, further research is needed to determine whether *E. coli* exhibits different roles and mechanisms in different intestinal segments.

Ulcerative Colitis (UC): In UC patients, there is a notable reduction in *Bifidobacterium* within the rectal MAM and an increase in *E. coli* [130]. Similarly, the colonic microbiota of UC patients shows decreased levels of *Bifidobacterium* and *Lactobacillus*, alongside increased *Bacteroides* and *E. coli* [133]. *E. coli* was observed clustering at the bottom of colonic crypts or within the lamina propria, indicating potential destruction of the intestinal mucosal barrier [133]. Dysbiosis in UC may trigger intestinal inflammation by disrupting this barrier. For example, *Bifidobacterium* enhance intestinal epithelial tight junctions via the Toll-like receptor-2 pathway through the NF-κB-independent mechanism, thereby preventing inflammation [134]. Conversely, *E. coli* secretes the proteolytic enzyme Vat-AIEC, which degrades mucin and reduces mucus viscosity, creating local cavities in the mucus layer [135]. Additionally, lipopolysaccharides (LPS) from E. coli can bind to TLR4 receptors on immune cells, promoting the release of pro-inflammatory factors and further inducing the inflammatory response [136]. These findings suggest that *E. coli, Bacteroides, Bifidobacterium,* and *Lactobacillus* are key microbes in UC development, with significant changes occurring in the colorectal region. However, the specific interactions and mechanisms of these microbiota within the colorectum require further investigation.

In summary, the microbiota in different intestinal segments have an important impact on the occurrence and progression of various diseases. Notably, even within the same disease, microbiota alterations may differ across intestinal segments. A comprehensive understanding of the dynamic changes and interactions of microbiota in various intestinal regions, and their association with diseases, is essential. This knowledge not only elucidates disease pathogenesis but also provides a theoretical foundation for precise microbiota regulation and the development of targeted treatment strategies.

## The mechanism of gut microbiota and its metabolites in regulating human health

In recent years, the role of GM and its metabolites in regulating human health has garnered significant research attention. The GM, which colonizes the human gut, has co-evolved with the host and is intricately linked with the external environment, establishing a dynamic ecological balance that participates in various physiological processes. However, the GM is susceptible to numerous factors such as age, environmental changes, hormone levels, medications (e.g. antibiotics), and dietary habits, etc. Dysregulation of the GM can disrupt homeostasis, potentially leading to a variety of clinical diseases [137, 138]. Extensive studies have shown that the GM mediates host-bacterial interactions mainly through small molecule metabolites produced during metabolic processes [139]. The six major metabolites of the GM include SCFAs, secondary bile acids (SBAs), trimethylamine N-oxide (TMAO), indole and its derivatives, endotoxin and vitamins. This review focuses on three critical metabolites—SCFAs, SBAs, and TMAO—and their related mechanisms in regulating physiological and pathological processes.

**Fig. 3:**
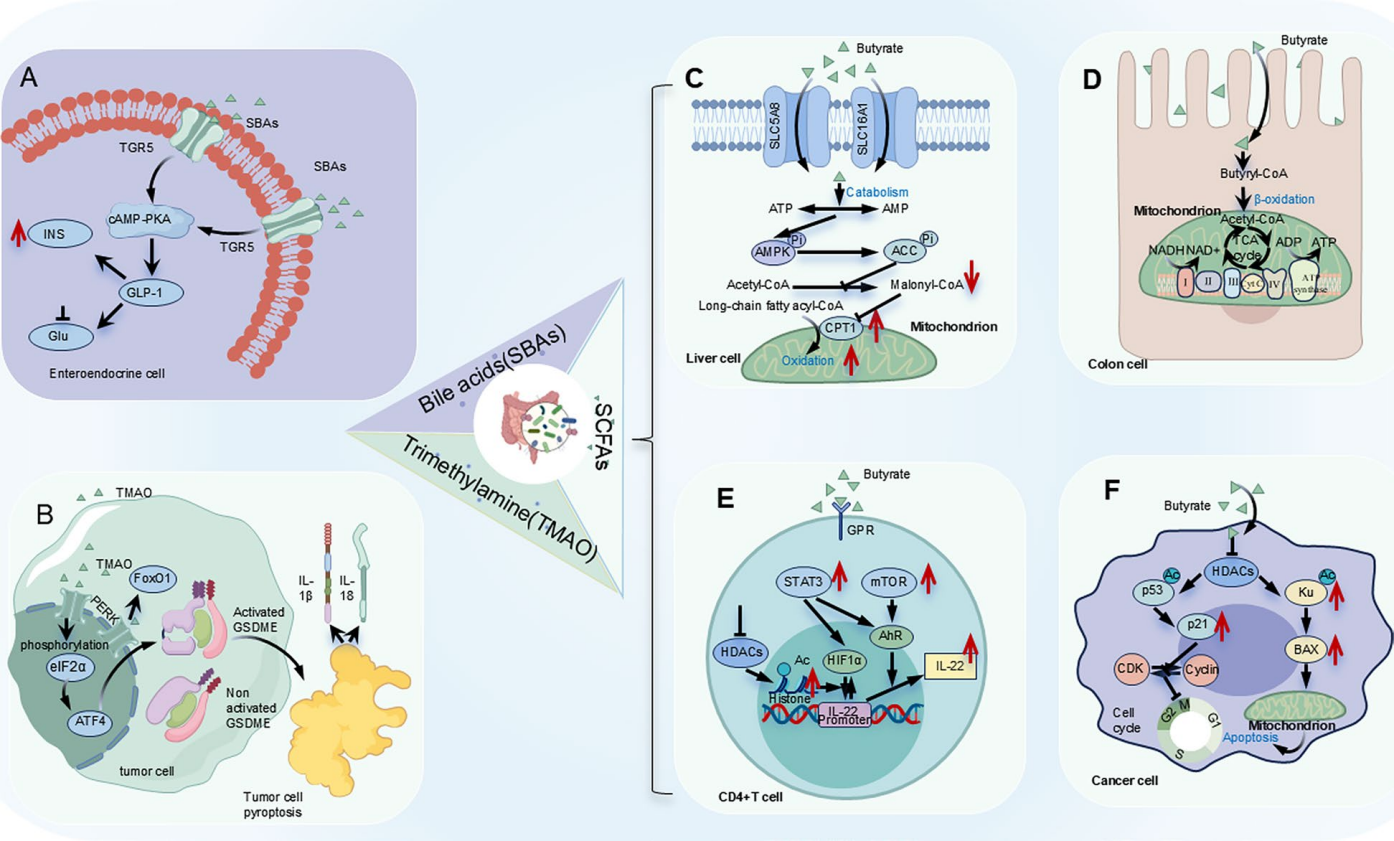
Mechanisms by which gut microbiota and their metabolites regulate body health. **A** Secondary Bile Acids (SBAs): Metabolites produced by gut microbiota activate GPR5 in intestinal endocrine cells by promoting the synthesis of glucagon-like peptide-1 (GLP-1). This induces insulin secretion and participates in regulating blood glucose balance. **B** Trimethylamine N-oxide (TMAO): A metabolic product of gut microbiota that activates the endoplasmic reticulum kinase PERK, inducing apoptosis of tumor cells mediated by the GSDME protein. This process releases inflammatory factors such as IL-1β and IL-18 into the tumor microenvironment, enhancing the infiltration and cytotoxic function of CD8^+^T cells. **C** Acetate and Butyrate: These short-chain fatty acids promote fatty acid oxidation and energy expenditure in mitochondria by activating 5′-AMP-activated protein kinase (AMPK). **D** Hydrochloric Acid Utilization: Colonic epithelial cells uptake and utilize hydrochloric acid as the primary energy source. **E** Butyrate and Immune Regulation: Butyrate activates GPR on CD4^+^T cells, regulates the expression of HIF1α and the aryl hydrocarbon receptor (AhR), promotes the production of large amounts of IL-22, and protects the body from intestinal infections and inflammation caused by intestinal injury. **F** Butyrate’s Anti-Tumor and Anti-Inflammatory Effects: Butyrate exerts these effects by inhibiting histone deacetylases (HDACs), suppressing cell proliferation and differentiation. It plays a crucial role in maintaining intestinal barrier function, mucosal immunity, and intestinal homeostasis.

Firstly, SCFAs, such as acetate, propionate and butyrate, are the main metabolites produced by the GM through the fermentation of carbohydrates and proteins. They are key molecules affecting metabolism and the immune system. Specifically, SCFAs not only provide energy to colonic epithelial cells but also play a pivotal role in the body’s overall energy metabolism [140]. For instance, colonic epithelial cells take up and utilize up to 95% of butyrate as the main energy source [141](Fig. 3C). Moreover, acetate and butyrate promote fatty acid oxidation and energy expenditure in mitochondria by activating 5′-AMP-activated protein kinase (AMPK) [142, 143](Fig. 3D). Beyond energy metabolism, SCFAs are also capable of regulating the immune response through the modulation of histone deacetylase (HDAC) activity and the activation of G protein-coupled receptor (GPR). For example, butyrate inhibits cell proliferation and differentiation by suppressing HDACs activity, thereby exerting anti-tumor and anti-inflammatory effects, and plays an important role in maintaining intestinal barrier function, mucosal immunity and intestinal homeostasis [141](Fig. 3E). Furthermore, butyrate activates GPRs on CD4⁺T cells, which in turn regulate the expression of hypoxia-inducible factor 1-alpha (HIF1α) and the aryl hydrocarbon receptor (AhR). This activation promotes the production of interleukin-22 (IL-22), enhancing the body’s defense against intestinal infections and inflammation caused by intestinal injury [144](Fig. 3F).

Secondly, SBAs, produced by GM metabolism, play an important role in regulating metabolism and maintaining intestinal barrier defense function. SBAs influence lipid and glucose metabolism by interacting with the farnesoid X receptor (FXR) and the Takeda G protein-coupled receptor 5 (TGR5). Specifically, SBAs activate TGR5 in enteroendocrine cells, promoting the synthesis of glucagon-like peptide-1 (GLP-1). GLP-1 induces insulin secretion and participates in the glucose homeostasis regulation [145] (Fig. 3A). Moreover, activation of FXR in the liver inhibits the SREBP1c-mediated lipogenesis pathway, thereby reducing triglyceride production [146]. In addition to metabolic regulation, SBAs inhibit the NF-κB signaling pathway by activating pregnane X receptor (PXR) and FXR. This inhibition reduces the production of inflammatory cytokine and intestinal permeability, thereby regulating intestinal immune function [147, 148].

Thirdly, TMAO is a primary metabolite generated by the GM through the metabolism of choline-containing compounds. It is involved in regulating glucose and lipid metabolism as well as the anti-tumor immune response. TMAO can induce gasdermin E (GSDME)-mediated tumor cell pyroptosis by activating the endoplasmic reticulum kinase PERK, leading to release inflammatory factors such as IL-1β and IL-18 into the tumor microenvironment (Fig. 3B). This process enhances the infiltration and cytotoxic function of CD8^+^T cells [149]. Moreover, TMAO selectively activates the PERK branch of the unfolded protein response, inducing the transcription factor FoxO1, a key regulator of metabolic disease in a PERK-dependent manner [150]. This activation upregulates the expression of gluconeogenic genes and downregulates the expression of glycolytic genes, which in turn influences glucose homeostasis. Meanwhile, it also upregulates the expression of ApoC-III in liver to increase plasma triglyceride, which promotes the occurrence and development of cardiovascular diseases such as atherosclerosis [151].

In conclusion, the GM and its metabolites are integral to various physiological and pathological processes, regulating overall health through complex and diverse mechanisms. Understanding these interactions not only provides a solid foundation for elucidating the specific roles and mechanisms of microbiota in different intestinal segments and diseases but also offers innovative avenues for developing precise therapeutic strategies based on GM modulation. This comprehensive knowledge enhances our ability to target the microbiome for improved diagnostic and treatment approaches across a wide range of health conditions.

## Challenges and perspectives

**Fig. 4:**
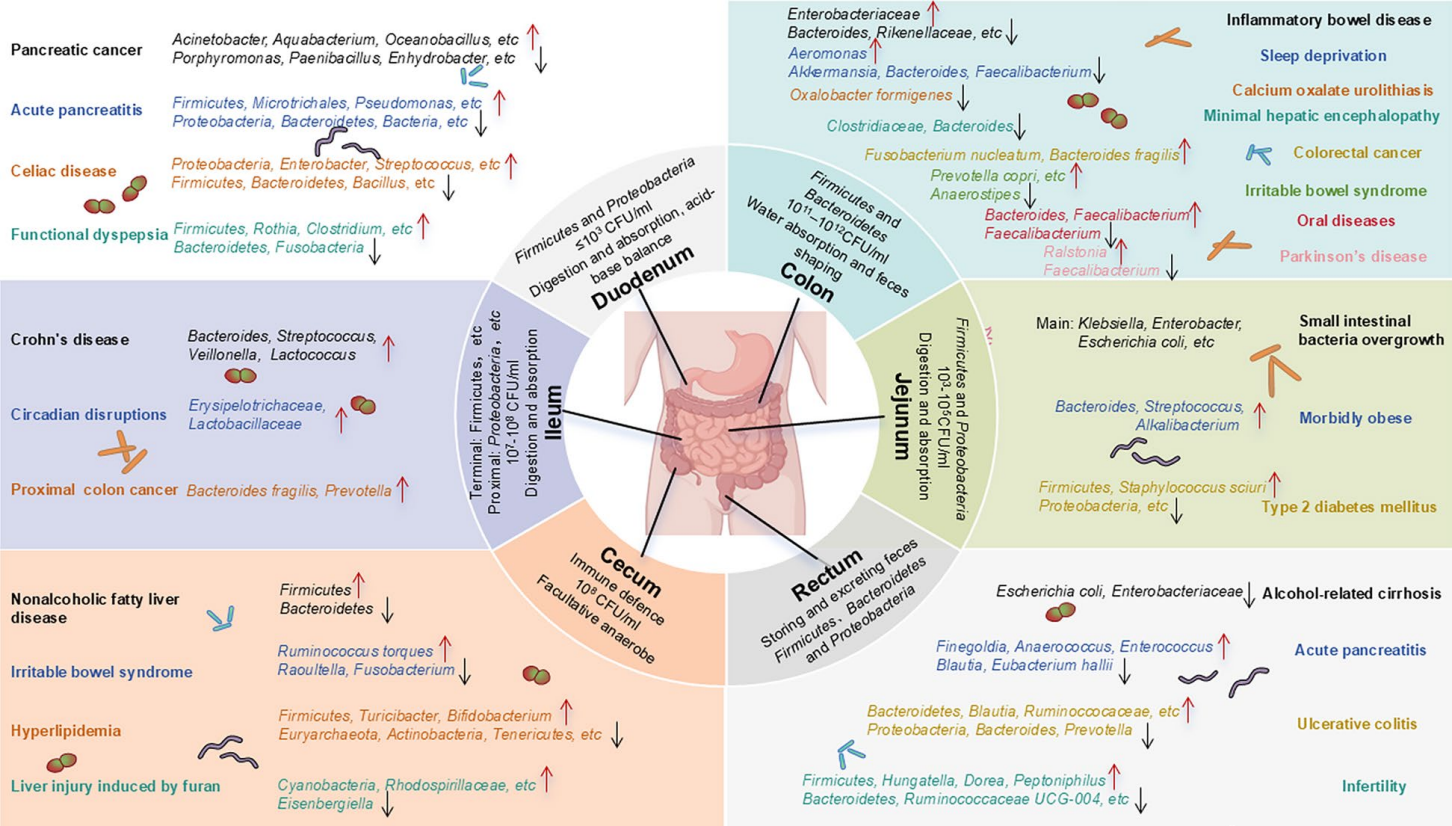
Physiological functions and microbiota characteristics of different intestinal segments, and the impact of microbiota in different intestinal segments on disease progression

**Fig. 5:**
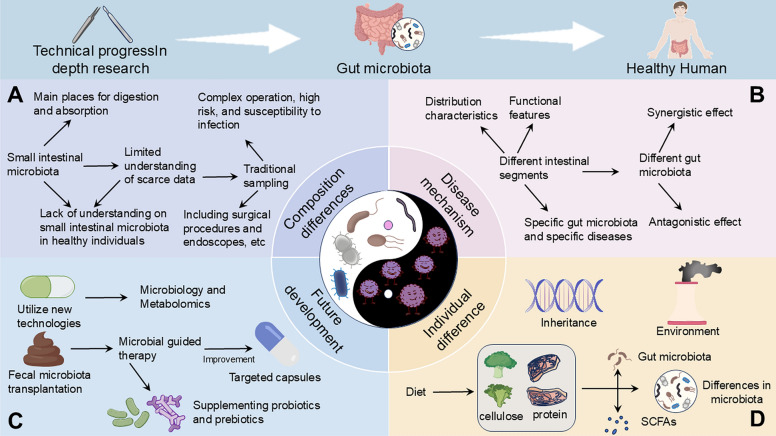
Challenges and Prospects in Comprehensive Gut Microbiome Research and the Development of Microbiome-Based Therapeutics. **A** Composition Differences: Limited sampling techniques have resulted in a scarcity of microbiome data from the small intestine, hindering a comprehensive understanding of its microbial composition. **B** Disease Mechanisms: There are distinct variations in microorganism distribution across different intestinal segments. Additionally, microbial communities exhibit both synergistic and antagonistic interactions that influence disease mechanisms. **C** Individual Differences: The composition and distribution of gut microbiota are influenced by a multitude of factors, including dietary habits, genetic predispositions, medication use, and existing health conditions, leading to significant inter-individual variability. **D** Future Developments: Advancements in microbiome and metabolomics technologies hold promise for enhancing research capabilities. However, challenges remain in implementing fecal microbiota transplantation and the direct supplementation of probiotics or prebiotics as effective treatment strategies

Despite the growing recognition of the intricate relationship between the GM and human health, several challenges hinder our comprehensive understanding and application of this knowledge. While extensive research has highlighted the critical role of GM in a wide range of diseases, most current studies predominantly focus on fecal samples (Fig. 4). This approach restricts our insight into the distribution and unique functions of microbiota across different intestinal segments. Addressing these limitations through in-depth exploration of GM distribution and mechanisms at various anatomical sites will significantly enhance our understanding of human gut microbial ecology and support the development of microbiome-based therapeutics for diverse diseases. These challenges underscore the urgent needs in the current research landscape and pave the way for future scientific inquiries, offering vast opportunities and possibilities (Fig. 5).

Firstly, the composition of microbiota varies significantly across different intestinal segments, and the mechanisms driving their formation remain largely unexplored. Notably, there is a scarcity of data on the microbiota in the small intestine— the primary site for food digestion and absorption— which likely has a profound impact on human health. The unique physiological structure of the small intestine poses challenges for sample collection, resulting in limited studies focused on the duodenum, jejunum, and ileum. Most existing research concentrates on colonic and fecal microbiota. Traditional sampling methods, such as surgery and endoscopy, are not only complex and risky but also prone to disturbing and infecting the microbial communities [152, 153]. Consequently, most available data on small intestinal microbiota derive from animal models or biopsy samples from patients with small intestinal diseases, severely restricting our understanding of the healthy small intestine microbiota.

To overcome these challenges, there is an urgent need for interdisciplinary collaborations aimed at developing novel sampling techniques that can accurately characterize the composition and function of the small intestinal microbiota. Emerging technologies, such as non-invasive sampling capsules of intestinal contents based on pH changes, offer a promising solution for more convenient and comprehensive microbial sampling across different intestinal segments. However, their applicability and accuracy in clinical settings require further validation [46, 47]. Continued development of innovative sampling methods is essential to elucidate the composition, distribution characteristics, and physiological functions of the small intestinal microbiota.

Secondly, the composition and distribution of the gut microbiome are influenced by a myriad of factors, including genetics, environment, emotions, diet and lifestyle. Racial and ethnic differences, for instance, play a significant role in shaping individual microbiota compositions. Studies have shown that individuals from the same ethnic background and geographical region tend to share similar GM characteristics [154]. This suggests that an individual's ethnic origin can serve as a marker for GM composition. However, long-term regional migrations can lead to the adaptation of different ethnic groups to new environmental conditions, resulting in the assimilation of their GM compositions [155]. These findings imply that both internal and external environmental factors are crucial determinants of GM diversity. When investigating the relationship between GM and diseases, it is essential to perform differential analysis of intestinal flora across diverse ethnic groups to understand the variations in health outcomes and disease susceptibilities among populations. Advanced sampling technologies, such as positioning capsules, can be utilized to collect and analyze GM composition along the entire intestinal tract [46]. This approach facilitates the establishment of personalized healthy microbial profiles, enabling early prediction of potential disease risks and the provision of individualized microbiome-based therapeutics.

Thirdly, the specific roles and mechanisms of microbiota in different intestinal segments concerning the body’s pathophysiological processes remain inadequately defined. The interaction between GM and various intestinal segments forms a complex network, where microbiota not only exhibit unique composition and functional characteristics but also engage in inter-segmental interactions influencing physiological and pathological states. Specifically, the gut microbiome communicates with other organs via multiple routes: bacterial metabolites, neurotransmitters, immune-inflammatory factors, vagus nerve and many others, which thus influences the state of human health through the establishment of the "gut-organ axis".Currently, clinical research predominantly utilizes fecal samples to explore GM-disease relationships, resulting in a limited understanding of how microbiota in specific intestinal segments contribute to diseases, such as Celiac Disease (CeD) and Colorectal Cancer (CC) [54, 156, 157]. Although certain studies have identified strong associations between specific intestinal segment microbiota and diseases, the underlying mechanisms have yet to be fully elucidated. Therefore, it is imperative to conduct more comprehensive research on the interactions between GM in different intestinal segments and their communication with host cells and tissues. However, correct and comprehensive evaluation of the gut microbiome is also a very difficult task. Employing advanced technologies, such as metagenomics and metabolomics, can analyze the composition and potential functions of a large number of microbes, and reflect how microbes interact with the host through their metabolites, which will enable a thorough investigation of microbiota functions across intestinal segments and their specific roles in disease onset and progression. This research will clarify the physiological functions and pathological effects of GM, offering new insights and approaches for the diagnosis and treatment of disease.

Fourthly, Microbiome-based therapeutics have emerged as a promising adjunct to conventional medical treatments, with continuous development and optimization in recent years. For instance, fecal microbiota transplantation (FMT) is an approach that restores microbial ecology by transplanting functional microbiota from healthy donors into patients’ guts to treat various diseases. However, the undefined composition of fecal samples poses risks of pathogen transmission, limiting the broader application of FMT [158]. Consequently, alternative microbiome-directed therapeutics, such as direct supplementation of probiotics or prebiotics which promote beneficial microbial proliferation, have gained attention. Clinical trials have demonstrated that oral supplementation with *Akkermansia muciniphila* effectively reduces inflammation-related blood markers and significantly improves insulin sensitivity in overweight or obese insulin-resistant individuals [159]. In addition to FMT and probiotics, antibiotics represent a conventional treatment modality that significantly impacts the gut microbiome. While antibiotics are essential for eliminating pathogenic bacteria, their broad-spectrum activity can disrupt the microbial balance, leading to reduced diversity and the emergence of antibiotic-resistant strains. This disruption varies across different intestinal segments due to the distinct microbial compositions and local environmental conditions, presenting challenges for microbiome recovery and maintenance post-antibiotic treatment. Developing precise interventions that target distinct intestinal regions is essential for enhancing therapeutic efficacy and minimizing systemic side effects. One potential solution involves designing targeted capsules tailored to the unique physicochemical environments or microbial characteristics of different intestinal segments. For example, enteric-coated capsules can ensure that probiotics or prebiotics are released in specific areas of the gastrointestinal tract, such as the colon, thereby optimizing their interaction with the local microbiota. Additionally, integrating advanced technologies like modern metagenomic and metabolomic methods can facilitate the differentiation of aerobic and anaerobic bacteria across various gut segments. These techniques enable a deeper understanding of the spatial dynamics of the gut microbiome, paving the way for the development of segment-specific therapies. Combining microbiome-directed treatments with other therapeutic strategies, such as dietary interventions or localized antibiotic treatments, may also offer synergistic benefits by modulating the microbiome in a controlled and targeted manner.

## Conclusion

As discussed, numerous studies have demonstrated significant differences in the distribution of GM across various intestinal segments, including the duodenum, jejunum, ileum, cecum, colon and rectum. This uneven distribution results in microbiota and their metabolites exerting distinct regulatory roles in the body’s physiological and pathological processes (Tables 1, 2).

Various factors contribute to gut microbiota dysbiosis, including diet, life cycle stages, medication treatments, and co-existing diseases. Dietary patterns, such as high-fat or high-fiber diets, can selectively promote or inhibit the growth of specific microbial communities in different gut regions. Life cycle stages, from infancy through old age, involve dynamic changes in microbiota composition influenced by developmental and hormonal shifts. Medication treatments, particularly antibiotics and proton pump inhibitors, can disrupt microbial balance by reducing microbial diversity and altering the abundance of key species in targeted gut segments. Additionally, co-existing diseases like diabetes, obesity, and inflammatory bowel disease create inflammatory environments that favor pathogenic microbes over beneficial ones, further exacerbating dysbiosis in specific intestinal areas.

Understanding how these factors differentially affect gut microbiota in specific segments of the gut strengthens the focus on spatial differences highlighted in this review. Consequently, in-depth exploration of the specific microbiota within certain intestinal segments and their mechanisms in disease contexts is crucial. Such investigations will lay an essential foundation for clinical applications, including microbiome-based diagnostics and precision therapeutics tailored to specific diseases.

The application of GM in treating conditions like inflammation and tumors remains a focal point in life sciences research. As the mechanisms by which specific microbiota in particular intestinal segments contribute to corresponding diseases become clearer, our understanding of the complex GM-disease relationship will deepen. This enhanced understanding will catalyze the development of early disease diagnostic tools and innovative therapeutic strategies based on GM modulation. Advancements in this field will herald a new era in promoting human health, providing more effective methods for disease prevention and treatment, and significantly improving patients' quality of life (Fig. 4).

## Data Availability

Not applicable.
